# Comorbidity patterns and risk of breast cancer: a case–control study exploring interaction with BMI using latent class analysis

**DOI:** 10.3389/fonc.2026.1908221

**Published:** 2026-08-24

**Authors:** Yaling Zhang, Bei Zhao, Xinchun Zhang, Na Cui, Lanhua Wu, Ling Chen

**Affiliations:** 1 Department of Nursing, The Affiliated Cancer Hospital of Xinjiang Medical University, Urumqi, China; 2 Department of Breast Surgery (Second Ward), The Affiliated Cancer Hospital of Xinjiang Medical University, Urumqi, China; 3 Department of Nephrology, First Hospital of Shanxi Medical University, Taiyuan, Shanxi, China; 4 The Affiliated Cancer Hospital of Xinjiang Medical University, Urumqi, China

**Keywords:** body mass index, breast cancer, comorbidity patterns, interaction effect, latent class analysis

## Abstract

**Background:**

Most studies to date have examined individual comorbidities in relation to breast cancer risk but have largely ignored that these conditions often cluster in the same person. Looking at comorbidity patterns rather than isolated diseases may therefore offer a better understanding of breast cancer risk.

**Methods:**

In this case–control study, latent class analysis (LCA) was performed in the pooled case–control sample based on 10 comorbidities/conditions, including diabetes mellitus, hypertension, coronary heart disease, benign breast disease, breast lump, nipple discharge, history of gynecological tumors, severe lobular hyperplasia of the breast, papillomatous lesions of the breast, and dysfunctional uterine bleeding. Multivariable unconditional logistic regression was then used to examine the association between the identified latent classes and breast cancer risk and to test for potential interaction with body mass index (BMI).

**Results:**

The LCA identified three comorbidity patterns in the pooled study sample: a low-comorbidity pattern, a metabolic comorbidity pattern, and a gynecologic–breast comorbidity pattern, accounting for 53.90%, 19.90%, and 26.20% of the study participants, respectively. Compared with the low-comorbidity pattern, both the metabolic comorbidity pattern (aOR = 2.55, 95% CI: 1.69–3.84) and the gynecologic–breast comorbidity pattern (aOR = 3.32, 95% CI: 2.25–4.89) were associated with higher breast cancer risk. Furthermore, a significant interaction was observed between BMI and the metabolic comorbidity pattern, suggesting that the association between this pattern and breast cancer risk varied across BMI categories.

**Conclusion:**

These findings suggest that a comorbidity-pattern-based approach can improve the understanding of breast cancer risk. Identifying specific comorbidity or clinical conditions, particularly the metabolic and gynecologic–breast comorbidity patterns, could inform personalized risk stratification and targeted prevention strategies for breast cancer. While BMI modified the association between the metabolic comorbidity pattern and breast cancer risk, no evidence of BMI-related effect modification was observed for the gynecologic–breast comorbidity pattern.

## 1 Background

Breast cancer is one of the most common malignancies among women worldwide, ranking first in incidence and remaining a leading cause of cancer-related mortality. According to GLOBOCAN 2022 estimates, approximately 2.3 million new cases of breast cancer were diagnosed globally, accounting for 11.6% of all cancers, with an estimated 666,000 deaths annually (1). The disease burden is particularly substantial in China. Among Chinese women, breast cancer has become the most commonly diagnosed malignancy, with approximately 357,200 new cases and 74,800 deaths, ranking first in incidence and third in cancer-related mortality. Notably, breast cancer in Chinese women shows a clear trend toward younger onset, with a peak incidence at 45–49 years, approximately 10 years earlier than that observed in Western populations (2). This earlier age at onset suggests that women may be exposed to relevant risk factors at a younger stage of life and may subsequently experience a longer disease course, including progression, recurrence, and long-term management burden (3). Early-onset breast cancer is often associated with prolonged survivorship and an increased risk of recurrence, which not only imposes substantial psychological and economic burdens on patients and their families but also presents significant challenges for healthcare systems.

With the increasing prevalence of chronic diseases, multimorbidity has become common in the general population. It reflects the accumulation and interaction of multiple chronic conditions within individuals and may contribute to breast cancer development through shared risk factors (4). Multimorbidity is not simply the sum of several diseases; rather, conditions tend to cluster in specific patterns and may jointly influence tumor initiation and progression through common pathophysiological pathways (5)—for example, chronic low-grade inflammation and insulin resistance associated with metabolic syndrome can inhibit apoptosis and enhance hypoxic adaptation in the tumor microenvironment, thereby promoting malignant transformation (6, 7). Thus, multimorbidity may reflect overall health status and also represent a potential risk factor for breast cancer.

Despite the growing recognition of its complexity, most epidemiological studies have focused on individual diseases, with limited attention to the overall comorbidity structure arising from multiple coexisting conditions. In real-world populations, diseases tend to cluster in specific patterns rather than occur randomly (8). The effects of these clusters on breast cancer risk are unlikely to be simply additive and may involve synergistic or interactive mechanisms (9). Therefore, analyses based solely on single diseases may not adequately capture the relationship between complex comorbidity structures and breast cancer risk.

To better characterize this complexity, a shift from a single-disease approach to a pattern-based framework is needed. Latent class analysis (LCA) is a data-driven method that identifies subgroups with similar comorbidity profiles based on individual-level data, allowing the underlying clustering structure of diseases to be characterized. Compared with traditional analyses, this approach provides a more comprehensive assessment of how comorbidity patterns relate to breast cancer risk (10).

Body mass index (BMI), an important indicator of metabolic status, has been consistently associated with breast cancer risk (11). In addition to its independent effect, BMI may modify the association between comorbidity patterns and breast cancer, acting as an effect modifier (12). However, the interaction between comorbidity patterns and BMI and its impact on breast cancer risk remain unclear.

In this case–control study, we applied LCA to identify comorbidity patterns based on data from cases and controls. We then examined their associations with breast cancer risk and evaluated their potential interaction with BMI, followed by sensitivity analyses to assess the robustness of the findings. By focusing on comorbidity patterns rather than individual diseases, this study aims to provide further insight into the complex risk structure of breast cancer and to inform risk stratification and prevention strategies.

## 2 Methods

### 2.1 Study population

**Figure 1:**
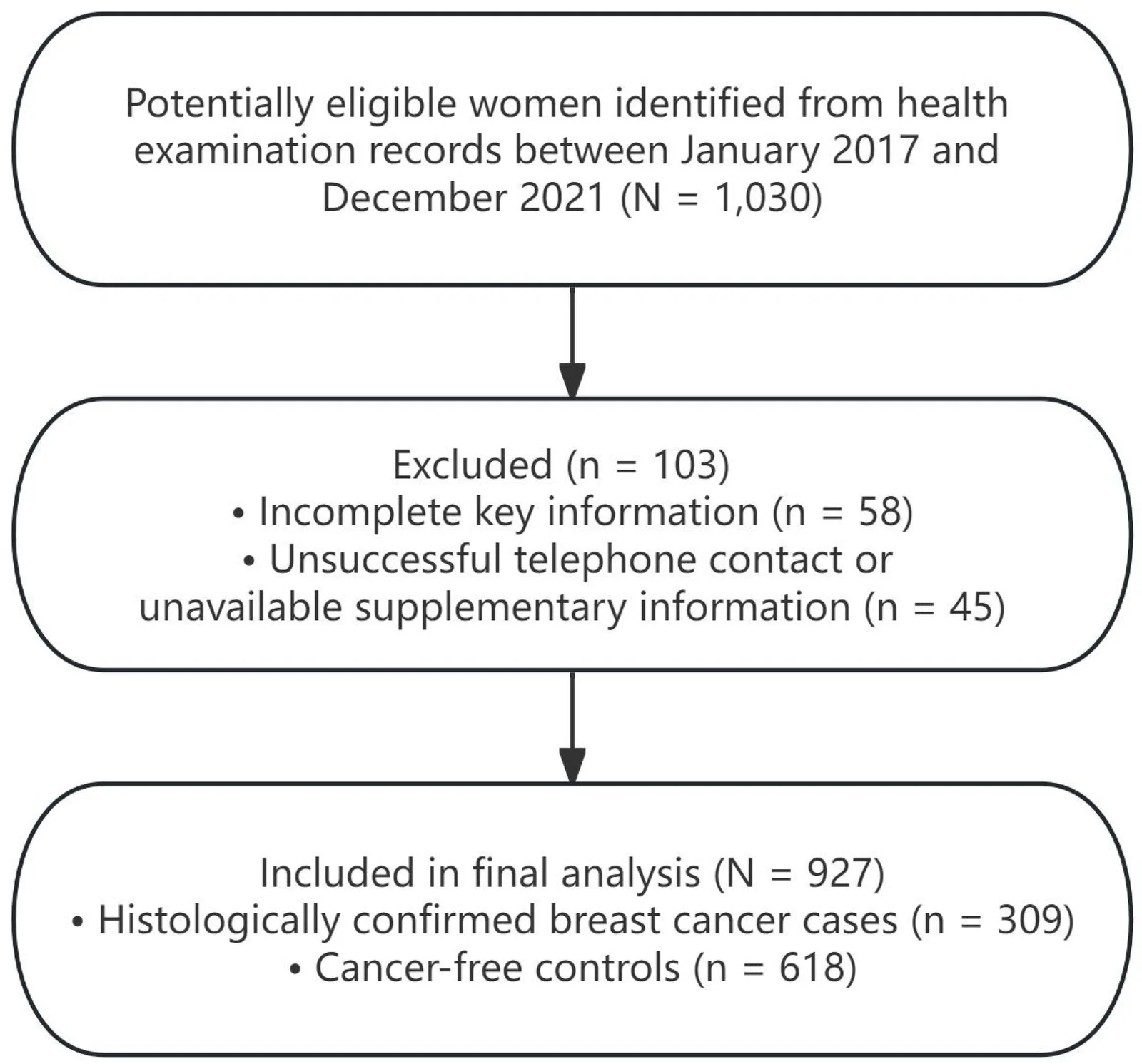
Flowchart of participant inclusion and exclusion. Controls were selected from cancer-free women who underwent health examinations during the same study period, resulting in a final case–control ratio of 1:2. No individual matching was performed.

This hospital-based case–control study was conducted among women who underwent health examinations at a tertiary specialty hospital between January 2017 and December 2021. Among 1,030 potentially eligible women identified from health examination records, 103 were excluded because of incomplete key information, unsuccessful telephone contact, or unavailable supplementary information. A total of 927 participants were included at a case–control ratio of 1:2, comprising 309 patients with histologically confirmed breast cancer and 618 cancer-free women who underwent routine health examinations at the Health Examination Center of the same hospital during the same period as the controls. The controls were not recruited from oncology outpatient or inpatient departments. Cancer-free status was determined based on health examination records, medical records, and telephone interview information, with no evidence or history of breast cancer or other malignant tumors. The 1:2 ratio referred to the case–control ratio rather than individual matching, and no individual matching was performed. The flowchart of participant inclusion and exclusion is shown in **Figure 1**.

### 2.2 Data collection

Data were obtained from two sources. First, the medical records of women who underwent health examinations between January 2017 and December 2021 were extracted from the hospital information system, including sociodemographic characteristics, lifestyle factors, BMI, reproductive and menstrual history, 10 comorbidity-related conditions, and family history of breast cancer. The comorbidities/conditions, including diabetes mellitus, hypertension, coronary heart disease, and breast- or gynecology-related conditions, were defined as pre-existing diseases or clinically relevant conditions before breast cancer diagnosis. These conditions were identified based on health examination records, self-reported previous medical history, physician-documented diagnoses, imaging findings, or pathological reports. The detailed definitions of all conditions are provided in **Supplementary Table S1**.

Second, telephone interviews were conducted to supplement missing or incomplete information not available in the hospital system, particularly lifestyle factors. Smoking history, alcohol consumption, and physical activity were collected via self-administered questionnaires as binary variables. For cases, exposure was defined as engagement in the relevant behavior for at least 1 year prior to breast cancer diagnosis; for controls, the reference period was the year before the health examination date (13–15). The interviews were performed by trained researchers using a structured questionnaire, and verbal informed consent was obtained at the beginning of each call. All data were anonymized prior to analysis.

### 2.3 Statistical analyses

The baseline characteristics of the case and control groups were compared using two-sample *t*-tests for continuous variables and chi-square tests for categorical variables. Latent class analysis (LCA) was performed in the pooled analytic sample of cases and controls based on the prevalence of 10 comorbidities or clinical conditions. Case–control status was not included as an indicator variable in the LCA model. Models with one to four classes were fitted, and the optimal number was selected using the Akaike information criterion (AIC), Bayesian information criterion (BIC), sample-size adjusted BIC (aBIC), and entropy as well as class size, model parsimony, model stability, and clinical interpretability. The identified classes were then labeled according to the distribution patterns of comorbidities or clinical conditions within each class. LCA was performed using the pooled sample of breast cancer cases and cancer-free controls to identify groups with different patterns of comorbidity-related conditions. After identifying the optimal latent class model, each participant was assigned to the latent class with the highest posterior probability. This most likely latent class membership was then used as the exposure variable in subsequent multivariable unconditional logistic regression analyses. Classification quality was assessed using entropy and average posterior probabilities.

Multivariable unconditional logistic regression models were used to evaluate associations between the identified latent classes and breast cancer risk, with the low-comorbidity pattern as the reference. The models were adjusted for potential confounders, including age, BMI, education level, age at menarche, age at first birth, menopausal status, history of estrogen use, family history of breast cancer, smoking status, alcohol consumption, and physical activity. Breastfeeding history, mammographic density, BRCA1/BRCA2 mutation status, and detailed dietary factors were not included in the models because these variables were not routinely or completely available from the hospital examination records or telephone interviews. To further assess effect modification by BMI, an interaction term between latent classes and BMI (categorized as <24 and ≥24 kg/m²) was included in the regression models. To test the robustness of the findings, a sensitivity analysis was conducted by treating BMI as a continuous variable in the multivariable analysis. We also repeated the LCA after excluding four breast-specific indicators, namely: benign breast disease, breast mass, severe lobular hyperplasia, and papillomatous lesions. The resulting classes were then included in the same multivariable logistic regression model. LCA was performed using Mplus version 8.3 (Muthén & Muthén, Los Angeles, CA, USA), and all other statistical analyses were conducted using SPSS version 26.0 (IBM Corp., Armonk, NY, USA). All tests were two-sided, and a *P*-value <0.05 was considered statistically significant.

### 2.4 Ethical approval

This study was approved by the Ethics Committee of the Affiliated Cancer Hospital of Xinjiang Medical University (approval no. K-2024201) and was conducted in accordance with the Declaration of Helsinki. Because this was a retrospective study and all data were anonymized prior to analysis, the requirement for written informed consent for medical record review was waived by the ethics committee. For the supplementary telephone interviews, verbal informed consent was obtained from each participant at the beginning of the call.

## 3 Results

### 3.1 Participants’ characteristics

A total of 927 women were included in this study, comprising 309 breast cancer cases and 618 controls. Compared with the control group, the patients in the case group were older (mean age: 42.58 ± 6.56 vs. 39.92 ± 12.48 years, *P* < 0.001) and had a higher mean BMI (24.81 ± 4.43 vs. 23.90 ± 4.70 kg/m², *P* < 0.001). Furthermore, a higher proportion of cases had attained a bachelor’s degree or higher (94.2% vs. 85.1%).

Regarding clinical and lifestyle characteristics, the case group was more likely to report a family history of breast cancer (18.4% vs. 7.8%, *P* < 0.001) and alcohol consumption (16.8% vs. 7.9%, *P* < 0.001) but reported a lower rate of physical activity (21.7% vs. 28.3%, *P* = 0.03). No significant differences were observed between the two groups regarding smoking status or history of estrogen use (**Table 1**).

**Table 1:** Comparison of baseline characteristics between cases and controls.

Characteristics	Cases (*n* = 309)	Controls (*n* = 618)	*P*-value
Age, years	42.58 ± 6.56	39.92 ± 12.48	<0.001
BMI, kg/m²	24.81 ± 4.43	23.90 ± 4.70	<0.001
Education level			<0.001
High school or below	18 (5.8%)	92 (14.9%)	
Bachelor’s degree or higher	291 (94.2%)	526 (85.1%)	
Age at menarche, years			<0.001
<12	54(17.5%)	42 (6.8%)	
≥12	255(82.5%)	576 (93.2%)	
Age at first birth, years			<0.001
<25	231 (74.8%)	562 (90.9%)	
25–34	34 (11.0%)	40 (6.5%)	
≥35	44 (14.2%)	16 (2.6%)	
History of estrogen use			0.067
No	296 (95.8%)	605 (97.9%)	
Yes	13 (4.2%)	13 (2.1%)	
Menopausal status			0.018
Premenopausal	260 (84.1%)	479 (77.5%)	
Postmenopausal	49 (15.9%)	139 (22.5%)	
Family history of breast cancer			<0.001
No	252 (81.6%)	570 (92.2%)	
Yes	57 (18.4%)	48 (7.8%)	
Smoking status			0.592
Never	306 (99.0%)	614 (99.4%)	
Ever	3 (1.0%)	4 (0.6%)	
Alcohol consumption			<0.001
Never	257 (83.2%)	569 (92.1%)	
Ever	52 (16.8%)	49 (7.9%)	
Physical activity			0.030
No	242 (78.3%)	443 (71.7%)	
Yes	67 (21.7%)	175 (28.3%)	

### 3.2 Identification of multimorbidity patterns

LCA was used to identify comorbidity patterns. **Table 2** shows the model fit indices. The AIC, BIC, and aBIC values decreased as the number of classes increased from one to four. Although the four-class model showed the lowest information criteria (AIC = 6,769.72, BIC = 6,977.50, aBIC = 6,840.93) and the highest entropy (0.832), it generated an additional small class accounting for 5.97% of the sample and did not provide a clearly distinct and clinically interpretable pattern beyond the three-class solution.

**Table 2:** Fit indices of latent class analysis models for multimorbidity patterns.

Model	AIC	BIC	aBIC	Entropy	LMRT (*P*-value)	BLRT (*P*-value)	Class distribution (%)
One-class	7,883.85	7,932.17	7,900.41	—	—	—	100.00
Two-class	7,296.55	7,398.02	7,331.32	0.705	<0.001	<0.001	48.00/52.00
Three-class	6,897.68	7,052.30	6,950.67	0.826	<0.001	<0.001	19.90/53.90/26.20
Four-class	6,769.72	6,977.50	6,840.93	0.832	0.0005	<0.001	5.97/32.27/19.71/42.16

The three-class model was selected as the final model based on a comprehensive consideration of model fit indices, entropy, class size, parsimony, model stability, and clinical interpretability. Although the four-class model showed lower AIC, BIC, and aBIC values and slightly higher entropy, it generated an additional small class accounting for 5.97% of the participants and did not provide a clearly distinct and clinically interpretable pattern beyond the three-class solution.

AIC, Akaike information criterion; BIC, Bayesian information criterion; aBIC, sample-size-adjusted Bayesian information criterion; LMRT, Lo–Mendell–Rubin adjusted likelihood ratio test; BLRT, bootstrapped likelihood ratio test.

Considering model fit indices, entropy, class size, model parsimony, model stability, and clinical interpretability, we selected the three-class model as the final model. This model had an entropy of 0.826, indicating acceptable classification quality. The average posterior probabilities for classes 1, 2, and 3 were 0.985, 0.894, and 0.931, respectively, further supporting acceptable classification accuracy. Additionally, both LMRT and BLRT supported the three-class model over the two-class model (*P* < 0.001). After excluding the four breast-specific indicators, the three-class solution remained the optimal model. The gynecologic–breast comorbidity pattern remained associated with breast cancer risk (**Supplementary Tables S2–S4**).

### 3.3 Characteristics of latent multimorbidity patterns

**Figure 2:**
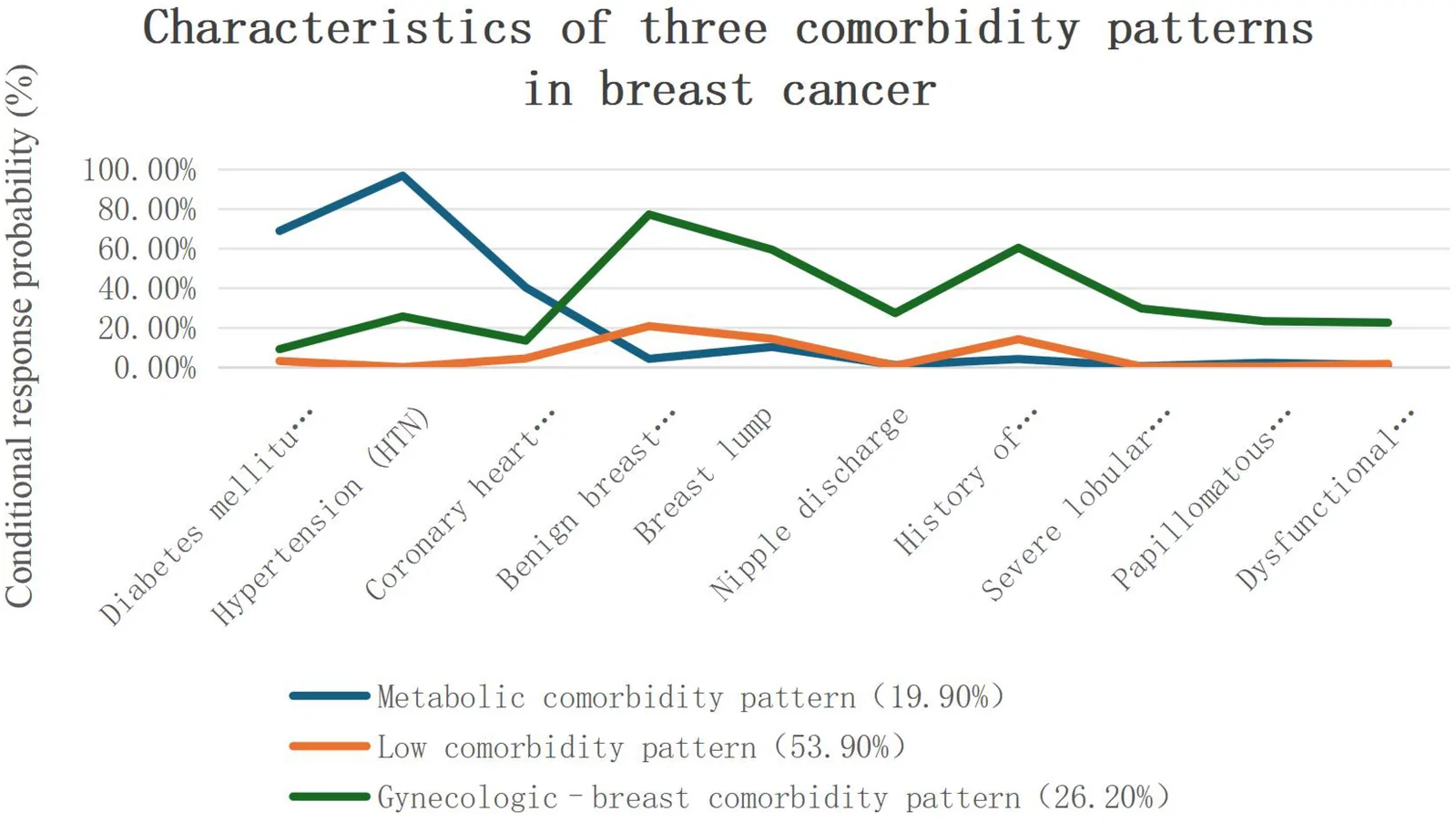
Characteristics of the three comorbidity patterns identified by latent class analysis.

Based on the conditional response probabilities of the 10 comorbidities (**Figure 2**), the three latent classes were characterized and labeled as follows: class 1 (19.90%), accounting for 19.90% of the study participants, was characterized by high probabilities of diabetes, coronary heart disease, and hypertension and was labeled the “metabolic comorbidity” pattern; class 2, accounting for 53.90% of the study participants, exhibited low probabilities across all evaluated comorbidities and was thus designated as the “low-comorbidity” pattern; finally, class 3 (26.20%), accounting for 26.20% of the study participants, demonstrated high probabilities of benign breast diseases, gynecologic tumors, and breast masses and was named the “gynecologic–breast comorbidity” pattern.

### 3.4 Association between comorbidity patterns and breast cancer risk

Using the low-comorbidity pattern as the reference, multivariable unconditional logistic regression analysis (**Table 3**) showed that both the metabolic comorbidity pattern and the gynecologic–breast comorbidity pattern were significantly associated with an increased risk of breast cancer, with adjusted odds ratios (aORs) of 2.55 (95% CI: 1.69–3.84, *P* < 0.001) and 3.32 (95% CI: 2.25–4.89, *P* < 0.001), respectively. In addition, age (aOR = 1.08, 95% CI: 1.06–1.10), BMI (aOR = 1.05, 95% CI: 1.02–1.09), undergraduate education or above (aOR = 2.61, 95% CI: 1.42–4.81), age at first birth ≥35 years (aOR = 2.59, 95% CI: 1.12–6.02), family history of breast cancer (aOR = 1.94, 95% CI: 1.21–3.13), and alcohol consumption (aOR = 2.52, 95% CI: 1.51–4.22) were also significantly associated with a higher risk of breast cancer. By contrast, age at menarche ≥12 years (aOR = 0.47, 95% CI: 0.29–0.77), age at first birth <25 years (aOR = 0.49, 95% CI: 0.27–0.88), postmenopausal status (aOR = 0.21, 95% CI: 0.12–0.35), and physical activity (aOR = 0.58, 95% CI: 0.40–0.84) were significantly associated with a lower risk of breast cancer.

**Table 3:** Association between comorbidity patterns and breast cancer risk using multivariable unconditional logistic regression.

Variable	aOR (95% CI)	*P*-value
Metabolic comorbidity pattern	2.55 (1.69–3.84)	<0.001
Gynecologic–breast comorbidity pattern	3.32 (2.25–4.89)	<0.001
Undergraduate education or above	2.61 (1.42–4.81)	0.002
Age at menarche ≥12 years	0.47 (0.29–0.77)	0.003
Age at first birth <25 years	0.49 (0.27–0.88)	0.017
Age at first birth ≥35 years	2.59 (1.12–6.02)	0.027
Estrogen use	2.31 (0.92–5.83)	0.075
Menopause	0.21 (0.12–0.35)	<0.001
Family history of breast cancer	1.94 (1.21–3.13)	0.006
Alcohol consumption	2.52 (1.51–4.22)	<0.001
Physical activity	0.58 (0.40–0.84)	0.004
Age	1.08 (1.06–1.10)	<0.001
BMI	1.05 (1.02–1.09)	0.004

The low-comorbidity pattern was used as the reference group. Adjusted odds ratios were estimated using multivariable unconditional logistic regression and were mutually adjusted for all of the variables listed in the table. Reference categories for categorical variables were defined as lower than undergraduate education, age at menarche <12 years, age at first birth 25–34 years, no estrogen use, premenopausal status, no family history of breast cancer, no alcohol consumption, and no physical activity. Age and BMI were included as continuous variables.

aOR, adjusted odds ratio; CI, confidence interval; BMI, body mass index.

### 3.5 Effect modification by BMI on the association between comorbidity patterns and breast cancer

To assess effect modification by BMI, interaction terms between BMI category and comorbidity patterns were included in the multivariable unconditional logistic regression model (**Table 4**). The interaction between the metabolic comorbidity pattern and BMI (≥24 kg/m²) was statistically significant (interaction aOR = 2.48, 95% CI: 1.08–5.69, *P* = 0.032), suggesting that the association between the metabolic comorbidity pattern and breast cancer risk differed by BMI category. To further clarify this interaction, stratified analyses were conducted according to BMI category (**Table 5**). In the BMI <24 kg/m² group, the metabolic comorbidity pattern was associated with increased odds of breast cancer compared with the low-comorbidity pattern (aOR = 1.74, 95% CI: 1.00–3.03, *P* = 0.049), whereas this association was more pronounced in the BMI ≥24 kg/m² group (aOR = 4.23, 95% CI: 2.20–8.15, *P* < 0.001). For the gynecologic–breast comorbidity pattern, the corresponding aORs were 3.48 (95% CI: 2.01–6.04, *P* < 0.001) in the BMI <24 kg/m² group and 3.12 (95% CI: 1.79–5.43, *P* < 0.001) in the BMI ≥24 kg/m² group. Consistently, no evidence of an interaction between the gynecologic–breast comorbidity pattern and BMI (≥24 kg/m²) was observed (interaction aOR = 0.96, 95% CI: 0.45–2.06, *P* = 0.918).

**Table 4:** Interaction between BMI and comorbidity patterns in relation to breast cancer risk using multivariable unconditional logistic regression.

Variable	aOR (95% CI)	*P*-value
Metabolic comorbidity pattern	1.78 (1.04–3.06)	0.037
Gynecologic–breast comorbidity pattern	3.37 (1.96–5.81)	<0.001
Undergraduate education or above	2.44 (1.33–4.50)	0.004
Age at menarche ≥12 years	0.48 (0.29–0.79)	0.004
Age at first birth <25 years	0.50 (0.27–0.92)	0.026
Age at first birth ≥35 years	2.81 (1.20–6.58)	0.017
Estrogen use	2.39 (0.94–6.03)	0.066
Menopause	0.21 (0.12–0.35)	<0.001
Family history of breast cancer	1.90 (1.17–3.08)	0.009
Alcohol consumption	2.33 (1.38–3.92)	0.001
Physical activity	0.57 (0.39–0.84)	0.004
Age	1.08 (1.05–1.10)	<0.001
BMI ≥24 kg/m²	1.41 (0.91–2.19)	0.125
Metabolic comorbidity pattern × BMI ≥24 kg/m²	2.48 (1.08–5.69)	0.032
Gynecologic–breast comorbidity pattern × BMI ≥24 kg/m²	0.96 (0.45–2.06)	0.918

The low-comorbidity pattern was used as the reference group. Adjusted odds ratios were estimated using multivariable unconditional logistic regression and were mutually adjusted for all of the variables listed in the table. Reference categories for categorical variables were defined as lower than undergraduate education, age at menarche <12 years, age at first birth 25–34 years, no estrogen use, premenopausal status, no family history of breast cancer, no alcohol consumption, and no physical activity. BMI was categorized as <24 and ≥24 kg/m² for interaction analysis, with BMI <24 kg/m² as the reference category. The interaction terms represent multiplicative interactions between comorbidity patterns and BMI ≥24 kg/m². Age was included as a continuous variable.

aOR, adjusted odds ratio; CI, confidence interval; BMI, body mass index. Sensitivity analysis.

**Table 5:** Stratified analysis of comorbidity patterns and breast cancer risk by BMI category.

BMI category	Comorbidity pattern	aOR (95% CI)	*P*-value
BMI <24 kg/m²	Low-comorbidity pattern	1.00	Reference
BMI <24 kg/m²	Metabolic comorbidity pattern	1.74 (1.00–3.03)	0.049
BMI <24 kg/m²	Gynecologic–breast comorbidity pattern	3.48 (2.01–6.04)	<0.001
BMI ≥24 kg/m²	Low-comorbidity pattern	1.00	Reference
BMI ≥24 kg/m²	Metabolic comorbidity pattern	4.23 (2.20–8.15)	<0.001
BMI ≥24 kg/m²	Gynecologic–breast comorbidity pattern	3.12 (1.79–5.43)	<0.001

The analyses were stratified by BMI category (<24 and ≥24 kg/m²). Within each BMI stratum, the low-comorbidity pattern was used as the reference group. Adjusted odds ratios were estimated using multivariable unconditional logistic regression with adjustment for education, age, age at menarche, age at first birth, estrogen use, menopausal status, family history of breast cancer, alcohol consumption, and physical activity.

aOR, adjusted odds ratio; CI, confidence interval; BMI, body mass index.

To assess the robustness of the findings, BMI was modeled as a mean-centered continuous variable instead of a categorical variable, and the multivariable unconditional logistic regression analysis was repeated (**Table 6**). The interaction between the metabolic comorbidity pattern and BMI (continuous) remained statistically significant (aOR = 1.14, 95% CI: 1.02–1.28, *P* = 0.020), suggesting that the association between the metabolic comorbidity pattern and breast cancer risk became stronger as BMI increased. In contrast, the interaction between the gynecologic–breast comorbidity pattern and BMI was not statistically significant (*P* = 0.829).

**Table 6:** Sensitivity analysis of the interaction between comorbidity patterns and mean-centered continuous BMI in relation to breast cancer risk using multivariable unconditional logistic regression.

Variable	aOR (95% CI)	*P*-value
Comorbidity patterns (class)		<0.001
Metabolic comorbidity pattern	2.75(1.80-4.20)	<0.001
Gynecologic–breast comorbidity pattern	3.33(2.26-4.91)	<0.001
Undergraduate education or above	2.60(1.40-4.82)	0.003
Age at menarche ≥12 years	0.46(0.28-0.76)	0.002
Age at first birth		<0.001
Age at first birth <25 years	0.52(0.28-0.96)	0.035
Age at first birth ≥35 years	2.90(1.23-6.81)	0.015
Estrogen use	2.42(0.96-6.14)	0.062
Menopause	0.21(0.12-0.36)	<0.001
Family history of breast cancer	1.95(1.20-3.16)	0.007
Alcohol consumption	2.37(1.41-3.98)	0.001
Physical activity	0.57(0.39-0.83)	0.003
Age	1.08(1.05-1.10)	<0.001
BMI, mean-centered	1.04(0.99-1.09)	0.158
Metabolic comorbidity pattern × BMI (mean-centered)	1.14(1.02-1.28)	0.020
Gynecologic–breast comorbidity pattern × BMI (mean-centered)	0.99(0.92-1.07)	0.829

The low-comorbidity pattern was used as the reference group. Adjusted odds ratios were estimated using multivariable unconditional logistic regression with adjustment for all of the variables listed in the table. Reference categories for categorical variables were below undergraduate education, age at menarche <12 years, age at first birth 25–34 years, no estrogen use, premenopausal status, no family history of breast cancer, no alcohol consumption, and no physical activity. BMI was mean-centered before constructing the interaction terms; therefore, the main effects of comorbidity patterns represent their associations with breast cancer risk at the sample mean BMI level rather than at BMI = 0.

aOR, adjusted odds ratio; CI, confidence interval; BMI, body mass index.

## 4 Discussion

In this study, three distinct comorbidity patterns were identified using LCA among breast cancer cases and controls: the metabolic comorbidity pattern, the gynecologic–breast comorbidity pattern, and the low-comorbidity pattern. In multivariable logistic regression analyses, with the low-comorbidity pattern as the reference, both the metabolic comorbidity pattern (aOR = 2.55, 95% CI: 1.69–3.84) and the gynecologic–breast comorbidity pattern (aOR = 3.32, 95% CI: 2.25–4.89) were positively associated with breast cancer risk. Further analyses showed a significant interaction between BMI category and the metabolic comorbidity pattern after adjustment for potential confounders (interaction aOR = 2.48, 95% CI: 1.08–5.69, *P* = 0.032). In the BMI-stratified analysis (**Table 5**), the association between the metabolic comorbidity pattern and breast cancer risk was more pronounced among women with BMI ≥24 kg/m² (aOR = 4.23, 95% CI: 2.20–8.15, *P* < 0.001) than among women with BMI <24 kg/m² (aOR = 1.74, 95% CI: 1.00–3.03, *P* = 0.049). In contrast, no evidence of an interaction was observed between BMI category and the gynecologic–breast comorbidity pattern (interaction aOR = 0.96, 95% CI: 0.45–2.06, *P* = 0.918). In the sensitivity analyses, BMI was modeled as a mean-centered continuous variable rather than as a categorical variable (**Table 6**). The interaction between the metabolic comorbidity pattern and mean-centered BMI remained statistically significant (interaction aOR = 1.14, 95% CI: 1.02–1.28, *P* = 0.020), suggesting possible BMI-related effect modification of the association between the metabolic comorbidity pattern and breast cancer risk. This finding was consistent with, but should be interpreted as supportive rather than confirmatory evidence for, the BMI-related interaction observed in the categorical BMI analysis.

In the covariate analysis, the present study not only observed associations consistent with several established risk factors for breast cancer but also suggested that these factors may not operate independently. They may instead interact through shared biological processes and jointly relate to breast cancer risk (16, 17). Age remained a significant risk factor, consistent with previous evidence showing that increasing age is closely associated with higher breast cancer risk and substantial age-related heterogeneity (18). Later age at menarche and earlier age at first birth were associated with lower risk, whereas older age at first birth was associated with increased risk, highlighting the close interplay between age and the female reproductive life course. Previous studies have also shown that age at menarche and age at menopause jointly determine the length of the reproductive period and partly reflect lifetime exposure to endogenous estrogen (19). These findings suggest that the effects of age and reproductive factors may converge on the cumulative impact of long-term endocrine exposure, which may be consistent with the stronger association between the gynecologic–breast comorbidity pattern and breast cancer risk. Family history, alcohol consumption, and physical activity may further contribute to risk accumulation through genetic susceptibility, hormone metabolism, and metabolic-inflammatory pathways and may correspond to the distinct risk characteristics of the gynecologic–breast and metabolic comorbidity patterns. In addition, evidence from the Korean population suggests marked differences in age-specific breast cancer incidence and screening detection patterns, indicating that age affects not only disease risk but also its epidemiological presentation (20). Against this background, risk-stratified individualized screening strategies have received increasing attention. Tailoring screening according to age and risk level may reduce breast cancer mortality while minimizing unnecessary false-positive recalls (21). These findings together support the possible involvement of two partly distinct biological processes suggested by the present study—a metabolism–inflammation-related process and a hormone-related process—and underscore the need for differentiated prevention and screening strategies for individuals with distinct risk profiles.

Previous studies have reported that the metabolic comorbidity pattern is associated with a 23% increase in breast cancer risk, possibly related to increased adipokine production in the context of metabolic syndrome (22). Our findings are broadly consistent, although the association observed in this study was slightly stronger, which may reflect a higher clustering of cardiovascular and metabolic conditions within this group. The link between obesity and breast cancer is likely mediated by multiple interrelated mechanisms, including adipose tissue inflammation, adipokine dysregulation, and alterations in the immune microenvironment. Under obese conditions, white adipose tissue can form crown-like structures that promote chronic inflammation and the release of pro-inflammatory cytokines. These cytokines not only stimulate breast epithelial cell proliferation but also induce aromatase expression, thereby increasing estrogen synthesis and establishing a positive feedback loop among obesity, inflammation, and estrogen signaling (23). Obesity may also promote tumor angiogenesis through IL-1β and contribute to carcinogenesis via epigenetic alterations, such as hypermethylation and silencing of tumor suppressor genes (24, 25). Taken together, these findings suggest that the metabolic comorbidity pattern may be linked to breast cancer risk through a metabolism–obesity–inflammation-related pathway. The observed association appeared to vary by BMI category, and the sensitivity analysis using mean-centered continuous BMI provided additional support for possible BMI-related effect modification. However, because this study was observational, these findings should be interpreted as suggestive rather than causal.

The present study found that the gynecologic–breast comorbidity pattern was significantly associated with breast cancer risk. However, no evidence of BMI-related effect modification was observed, although this finding should be interpreted cautiously. This pattern, characterized by a high prevalence of benign breast disease, gynecologic tumors, and breast lumps, suggests the involvement of shared hormonal pathways. Early menarche, late menopause, nulliparity, and older age at first birth were associated with increased risk, whereas earlier childbearing appeared to be protective. These findings are consistent with previous reports indicating that each 1-year delay in age at menarche is associated with an approximately 5% reduction in breast cancer risk, each 1-year delay in age at menopause with an approximately 3% increase in risk, and age at first birth after 35 years with a substantially higher risk compared with early first birth (26).While estrogen has traditionally been considered a key driver of breast cancer, emerging evidence suggests that the breast and female reproductive system share common hormonal pathways and may be jointly affected by endocrine dysregulation (27). Estrogen may enhance progesterone signaling by upregulating progesterone receptor expression, whereas progesterone itself may play a more direct role in promoting breast epithelial cell proliferation, particularly during the luteal phase (28). In addition, molecular heterogeneity in breast cancer implies varying degrees of hormone dependence across subtypes (29). The gynecologic–breast comorbidity pattern observed in this study may therefore reflect a clinical manifestation of such shared hormonal dysregulation. From a clinical perspective, individuals with this pattern may represent a high-risk group and may warrant closer follow-up and risk-adapted screening in health checkup settings.

By applying LCA to the pooled case–control sample and further examining BMI interactions, this study identified distinct comorbidity patterns associated with breast cancer risk and suggested two possible biological processes: a metabolism–inflammation-related process and a hormone-related process. However, several limitations should be noted. As an observational study, it was not possible to determine the temporal or causal relationship between comorbidity patterns and breast cancer. Some breast-specific indicators in the main LCA model may overlap with premalignant conditions or early clinical findings. Therefore, our findings should be interpreted as risk stratification in a health checkup population rather than evidence of causal effects. Although the classification quality was acceptable, the regression analyses used the most likely class assignment, which may not fully account for classification uncertainty and could attenuate the estimated associations. Future studies using BCH or maximum-likelihood three-step methods are needed to validate these findings. Selection bias cannot be excluded because this was a single-center hospital-based case–control study conducted in a tertiary specialty hospital. Although cases and controls were selected from the same hospital during the same period, the cancer-free controls were health examination attendees and may differ from the general female population in health awareness, healthcare-seeking behavior, and baseline disease risk. Therefore, the generalizability of our findings should be interpreted with caution. In addition, because LCA was performed in a case-enriched case–control sample, the proportions of the identified comorbidity patterns reflect the study sample and should not be interpreted as population-level estimates. Lifestyle factors, including smoking, alcohol consumption, and physical activity, were collected as self-reported binary variables through telephone interviews, without detailed information on exposure intensity, frequency, or amount. Additionally, several established breast cancer risk factors, including breastfeeding history, mammographic breast density, BRCA1/BRCA2 mutation status, and dietary factors, were unavailable in our dataset and could not be included in the analysis. Moreover, molecular subtype information was only available for breast cancer cases and was therefore insufficient for subtype-specific analyses. Future studies incorporating comprehensive risk factor profiles and complete immunohistochemical data are warranted to further clarify the relationship between comorbidity patterns and breast cancer risk. Although the BMI-stratified analysis and the sensitivity analysis using mean-centered continuous BMI supported possible BMI-related effect modification, these findings should be interpreted cautiously because subgroup analyses may have limited precision. Similarly, the non-significant interaction for the gynecologic–breast comorbidity pattern should not be interpreted as evidence of BMI independence. Larger multicenter studies are needed to confirm the interaction between the metabolic comorbidity pattern and BMI.

Breast cancer risk appears to be closely associated with specific comorbidity patterns. Both the metabolic comorbidity pattern and the gynecologic–breast comorbidity pattern were associated with an increased risk of breast cancer; however, the underlying biological processes may differ. The association between the metabolic comorbidity pattern and breast cancer risk appeared to be more pronounced among women with BMI ≥24 kg/m² and was supported by both the categorical interaction model and the sensitivity analysis using mean-centered continuous BMI. In contrast, no evidence of BMI-related effect modification was observed for the gynecologic–breast comorbidity pattern, which may primarily reflect hormone-related pathways. By integrating latent class analysis with BMI interaction assessment, this study provides a novel epidemiological perspective for understanding heterogeneity in breast cancer risk across different comorbidity profiles.

These findings provide a novel perspective on risk stratification and precision prevention strategies in breast cancer. For individuals with the metabolic comorbidity pattern, greater emphasis should be placed on weight control and metabolic management. In contrast, for those with the gynecologic–breast comorbidity pattern, priority should be given to reproductive endocrine evaluation and targeted early screening.

## Data Availability

The raw data supporting the conclusions of this article will be made available by the authors, without undue reservation.
