# Supplementary material for: Comorbidity patterns and risk of breast cancer: a case–control study exploring interaction with BMI using latent class analysis
Source: Front Oncol. 2026 Aug 24;16:1908221. doi: 10.3389/fonc.2026.1908221 (PMC13546989; doi:10.3389/fonc.2026.1908221)
Supplement: Supplementary file 1 [file Table1.docx]

**Supplementary Table S1. Definitions of comorbidity-related conditions included in latent class analysis**

| Variable | Definition |
| --- | --- |
| Diabetes mellitus (DM) | Previous physician-documented diagnosis of diabetes mellitus recorded in the medical records. |
| Hypertension (HTN) | Previous physician-documented diagnosis of hypertension recorded in the medical records. |
| Coronary heart disease (CHD) | Previous physician-documented diagnosis of coronary heart disease recorded in the medical records. |
| Benign breast disease (BBD) | Previous clinical or pathological diagnosis of benign breast disease documented in the medical records. |
| Breast lump | Presence of a breast mass documented by clinical examination or imaging examination records. |
| Nipple discharge | Abnormal nipple discharge documented in clinical records. |
| History of gynecological tumors | Previous physician-documented diagnosis of gynecological tumors recorded in the medical records. |
| Severe lobular hyperplasia of the breast | Pathological diagnosis of severe lobular hyperplasia documented in pathological reports. |
| Papillomatous lesions of the breast | Pathological diagnosis of papillomatous lesions documented in pathological reports. |
| Dysfunctional uterine bleeding (DUB) | Previous clinical diagnosis of dysfunctional uterine bleeding recorded in the medical records. |

**Supplementary Table S2. Fit indices of LCA models after excluding four breast-specific indicators**

| Model | AIC | BIC | aBIC | Entropy | LMRT P-value | BLRT P-value | Class distribution (%) |
| --- | --- | --- | --- | --- | --- | --- | --- |
| 1-class model | 4692.895 | 4721.886 | 4702.831 | — | — | — | 100.00 |
| 2-class model | 4281.660 | 4344.475 | 4303.189 | 0.939 | <0.001 | <0.001 | 27.17 / 72.84 |
| 3-class model | 4146.418 | 4243.057 | 4179.539 | 0.853 | <0.001 | <0.001 | 22.35 / 12.50 / 65.15 |
| 4-class model | 4118.545 | 4249.008 | 4163.259 | 0.840 | 0.0018 | <0.001 | 15.61 / 60.79 / 1.15 / 22.45 |

Note: AIC, Akaike information criterion; BIC, Bayesian information criterion; aBIC, sample-size adjusted Bayesian information criterion; LMRT, Lo-Mendell-Rubin likelihood ratio test; BLRT, bootstrap likelihood ratio test. Class distribution refers to the model-estimated class proportions. The four excluded breast-specific indicators were benign breast disease, breast lump, severe lobular hyperplasia, and papillomatous lesions. Although the 4-class model showed lower AIC and aBIC, it generated a very small class comprising only 1.15% of the sample, suggesting potential over-extraction. Therefore, the 3-class model was selected for the sensitivity analysis based on BIC, acceptable entropy, adequate class size, parsimony, and clinical interpretability.

**Supplementary Table S3. Item-response probabilities of the selected 3-class model after excluding four breast-specific indicators**

| Indicator | Class 1: Metabolic comorbidity pattern | Class 2: Gynecologic-related clinical status pattern | Class 3: Low comorbidity pattern |
| --- | --- | --- | --- |
| TDM | 0.627 | 0.131 | 0.033 |
| EH | 0.984 | 0.321 | 0.000 |
| CHD | 0.364 | 0.218 | 0.047 |
| ND | 0.015 | 0.428 | 0.032 |
| HGT | 0.075 | 0.838 | 0.184 |
| AUB | 0.020 | 0.447 | 0.015 |
| Class proportion (%) | 22.35 | 12.50 | 65.15 |

Note: Values represent the conditional probability of endorsing category 2 for each indicator within each latent class. Class 1 was characterized by high probabilities of TDM, EH, and CHD and was therefore named the metabolic comorbidity pattern. Class 2 was characterized by relatively high probabilities of ND, HGT, and AUB and was named the gynecologic-related clinical status pattern. Class 3 had generally low probabilities across indicators and was named the low comorbidity pattern.

**Supplementary Table S4. Sensitivity analysis of the association between latent classes and breast cancer risk after excluding four breast-specific indicators using multivariable unconditional logistic regression**

| Variable | aOR (95% CI) | P-value |
| --- | --- | --- |
| Metabolic comorbidity pattern | 1.46 (1.00–2.15) | 0.051 |
| Gynecologic-related clinical status pattern | 3.34 (2.01–5.54) | <0.001 |
| Undergraduate education or above | 3.08 (1.67–5.68) | <0.001 |
| Age at menarche ≥12 years | 0.44 (0.27–0.71) | <0.001 |
| Age at first birth <25 years | 0.33 (0.18–0.58) | <0.001 |
| Age at first birth ≥35 years | 1.67 (0.72–3.86) | 0.232 |
| Estrogen use | 1.87 (0.75–4.65) | 0.179 |
| Menopause | 0.25 (0.14–0.42) | <0.001 |
| Family history of breast cancer | 2.09 (1.31–3.34) | 0.002 |
| Alcohol consumption | 2.54 (1.54–4.21) | <0.001 |
| Physical activity | 0.60 (0.41–0.86) | 0.006 |
| Age | 1.07 (1.03–1.11) | <0.001 |
| BMI | 1.07 (1.05–1.09) | <0.001 |

Note: The low comorbidity pattern was used as the reference group. Adjusted odds ratios were estimated using multivariable unconditional logistic regression and were mutually adjusted for all variables listed in the table. The four excluded breast-specific indicators were benign breast disease, breast mass, severe lobular hyperplasia, and papillomatous lesions. Age and BMI were entered as continuous variables.
